# Effect of Storage Temperatures on Physico-Chemicals, Phytochemicals and Antioxidant Properties of Watermelon Juice (*Citrullus lanatus*)

**DOI:** 10.3390/metabo12010075

**Published:** 2022-01-13

**Authors:** Nur Shafinaz Mohamad Salin, Wan Mazlina Md Saad, Hairil Rashmizal Abdul Razak, Fatimah Salim

**Affiliations:** 1 Centre of Medical Laboratory Technology, Faculty of Health Sciences, Puncak Alam Campus, Universiti Teknologi MARA (UiTM) Selangor Branch, Puncak Alam 42300, Selangor, Malaysia; nshafinazms@gmail.com; 2 Department of Radiology, Faculty of Medicine and Health Sciences, Universiti Putra Malaysia (UPM), Serdang 43400, Selangor, Malaysia; rashmizal@upm.edu.my; 3 Atta-ur-Rahman Institute for Natural Product Discovery (AuRIns), Puncak Alam Campus, Universiti Teknologi MARA (UiTM) Selangor Branch, Puncak Alam 42300, Selangor, Malaysia; fatimah2940@uitm.edu.my; 4 Centre of Foundation Studies, Dengkil Campus, Universiti Teknologi MARA (UiTM) Selangor Branch, Dengkil 43800, Selangor, Malaysia

**Keywords:** watermelon juice, storage, phytochemical, antioxidant

## Abstract

Watermelon (*Citrullus lanatus*) consists of high moisture content and is favoured for its juice products. The popular fruit has a tempting taste, sweet aroma and attractive flesh colour. It is enriched with phytochemicals and antioxidant properties that are beneficial to human health. Due to convenience, the majority of individuals are likely to consume watermelon juice. However, little is known about the fruit juice storage and temperatures that may affect its beneficial properties. This study investigated the effect of storage temperature at room temperature, refrigerator cold, refrigerator freeze and freeze-dried, and analyzed the juice physico-chemicals (weight loss, pH, ash, moisture, total soluble solid, browning and turbidity), phytochemicals (total phenolic, total flavonoid, lycopene and β-carotene) and antioxidant scavenging activities during 9 days of storage. The results showed that watermelon juice was affected by storage temperatures and conditions with significant changes in physico-chemical appearance and decrease in total phytochemical content, thus consequently affecting their antioxidant activities during 9 days of storage. Although fresh watermelon juice can be consumed for its high nutritional values, freeze-drying is the preferable technique to retain its benefits and to delay juice degradation.

## 1. Introduction

Watermelon, or *Citrullus lanatus*, belongs to the Cucurbitacea family and is highly consumed due to its health benefits. In fact, the fruit is known as the king of summer fruit that quenches summer thirst [1]. It can be consumed directly by eating the flesh or as juice. Due to its high moisture content, the most preferred and popular product of watermelon is the juice [2], mainly due to its sweetness, refreshing taste and attractive colour intensity [3]. As a consumer interest, the healthy beverages have grown over the years, and the juice market too is rapidly expanding [4]. Hence, this has created a huge demand for watermelon juice production that is perpetuated not only by the colour, taste and aroma, but also the bioactive compounds such as carotenoids (lycopene and β-carotene), flavonoids, phenolics, amino acids (L-citrulline and L-arginine) and vitamins (A and C) that were found to be significant [5,6,7]. 

Watermelon is a non-seasonal fruit that has been cultivated abundantly in Malaysia and other tropical regions [1]. Watermelon contains high water content of approximately 93% of its weight, hence the name “water”, and melon for the fruit’s morphological characteristics of shape, sweetness taste and pulpy flesh [1]. Furthermore, botanists refer to watermelon as “pepo”; a fruit having a thick rind and fleshy centre [8]. A study by Saad et al. (2020) categorized watermelon into three different parts; flesh (68%), peel (30%), and seed (2%) [1]. Watermelon fruit yields 55.3% juice, 31.5% rind and 10.4% pomace [8], and the consumption of watermelon juice is reported to be a good source of bioactive properties that are significantly beneficial to human health [9].

Therefore, storage of fruit juice is the crucial part in maintaining its physical and bioactive activities which would reflect the juice quality and its acceptability. Much effort has been made to store watermelon juice to reduce juice degradation, increase shelf life and retain nutritional compositions [10]. To keep the refreshing taste of watermelon juice, consumers tend to store it in various conditions that include ambient temperature, refrigerator at 4 °C or frozen at −10 to −20 °C [11,12]. Other methods were also performed to maintain the overall quality of aroma, colour, taste and phytochemical properties of watermelon juice that includes high-pressure treatment, high-pressure carbon dioxide treatment, pulse electric field treatment, ultrasound treatment, high hydrostatic pressure, freeze-dried, pasteurization and sterilization [2,11,13]. However, the mentioned techniques were intermittent, expensive and not easily used by consumers [13].

A previous study demonstrated that watermelon juice stored at 4 °C presented a shelf life of 4 days, and was unacceptable for consumption after day 7 [14]. The study by Queirós et al., 2014 presented a shelf life of fruit juice stored at ambient temperature of 20–25 °C but is less than 8 h [15]. For this reason, temperature seems to be a key factor in fruit juice storage. In addition, study on the effects of storage temperature on watermelon juice is only partially discovered. Watermelon juice is sensitive to ambient changes in temperature, light, oxygen and ion [11]. A study by Liu et al., 2018 mentioned that watermelon quality and flavour were affected by light, thermal and time exposure towards atmosphere and surroundings [2]. During storage, the juice undergoes physical changes of colour, cloudiness, sour smell and taste that indicate the first evaluation made in determining the juice quality [15]. This physical evaluation reflects phytochemical and bioactive constituent presented in watermelon juice. Not by only the unacceptable physical evaluation of juice, the phytochemical and antioxidant properties are able to be destroyed and bioavailability reduced, hence reducing the health impact [16]. 

In this study, the effects of temperature on physico-chemical, phytochemical and antioxidant properties of watermelon juice (*Citrullus lanatus*) during storage at room temperature, refrigerator cold, refrigerator freeze and freeze-dried for 9 days were determined. Room temperature, refrigerator cold and refrigerator freeze storage were chosen as they are the frequent methods used by consumers to retain juice freshness [11,12]. Furthermore, previous study also mentioned that freeze-drying retained more bioactive compounds and is one of the preferable methods of food products [11]. However, the effect on watermelon juice during storage at the respective conditions were partially explored.

## 2. Results and Discussion

### 2.1. Weight Loss (%)

**Figure 1:**
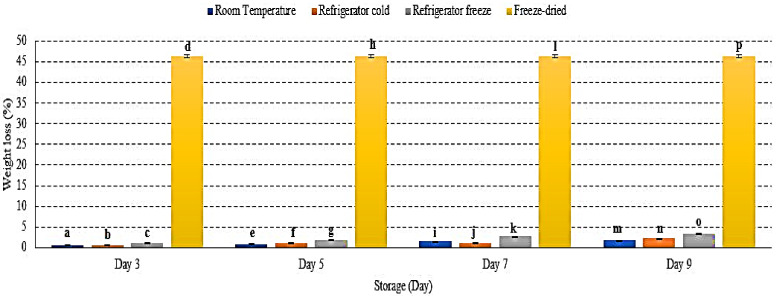
The weight loss of watermelon at different storage temperatures during 9 days of storage. Different letters of a, b, c, d, etc. indicate significant differences between samples *p <* 0.01.

Weight loss refers to the loss of water content in fruit [17]. There was an increase of watermelon weight loss (%) in all storage with significant differences observed at *p* < 0.01 (Figure 1). Freeze-dried watermelon lost the most weight due to the lyophilization process during freeze-drying [18]. Room temperature storage displayed the lowest percentage of weight loss during 9 days of storage (Figure 1). Increased weight loss indicates an increased respiration rate and metabolic processes, which were prominently activated during cold storage [19]. In addition, the gradient difference of vapor pressure between the surrounding and fruit tissue plays an important role in weight loss [20]. Studies presented the percentage weight loss as up to 75% for freeze-dried longan fruit [18], 14% for mango fruit stored at room temperature (13 ± 1 °C) [20], and 10.67% for strawberry fruit stored in a refrigerator at 6 ± 1 °C, which further supported the present data [19]. Hence, this study demonstrated the loss of watermelon weight advent with storage conditions that include temperature, vapour pressure and humidity [19,20].

### 2.2. pH 

**Figure 2:**
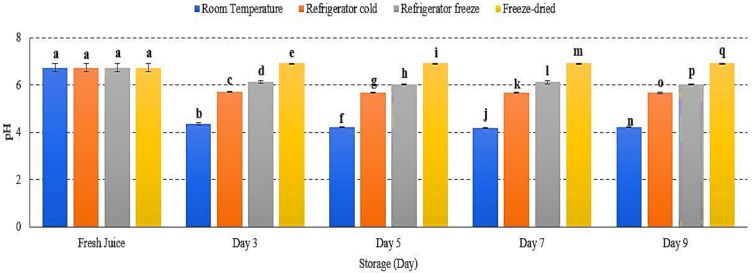
The pH values of watermelon juice during 9 days of storage at different storage temperatures. Different letters indicate significant differences between samples *p <* 0.01.

pH was used to measure the degree of acidity in fruit juice and reflect fruit taste, smell and aroma [21]. The pH of watermelon juice decrease in room temperature, refrigerator cold and refrigerator freeze with slight pH increase was evaluated in freeze-dried juice *p* < 0.01 (Figure 2). A major decrease in pH was observed in room temperature storage (Figure 2), indicating an increased growth of mesophilic bacteria in optimum conditions. The acidic condition allows acid-tolerant pathogenic bacteria to grow such as *Salmonella* spp., *Staphylococcus aureus*, *Escherichia coli* and *Listeria monocytogenes* [22,23]. These pathogenic bacteria are able to survive and induce the development of spores and toxin, resulting in increased degree of acidity [22]. Meanwhile, an increase in pH may indicate the reaction of acid hydrolysis during the freeze-drying procedure, in which the endogenous α-amylase bond is inactivated and causes damage in starch granules, which later results in the transformation of acid hydrolysis into reducing sugar [24,25]. Increased accumulation of reducing sugar eventually reduces the degree of acidity in fruit [24,26]. Hence, the data presented an increase in pH of watermelon juice attributed to transformation of acid hydrolysis, while a decrease in pH due to a rise in toxin production. 

### 2.3. Ash Content (%)

**Figure 3:**
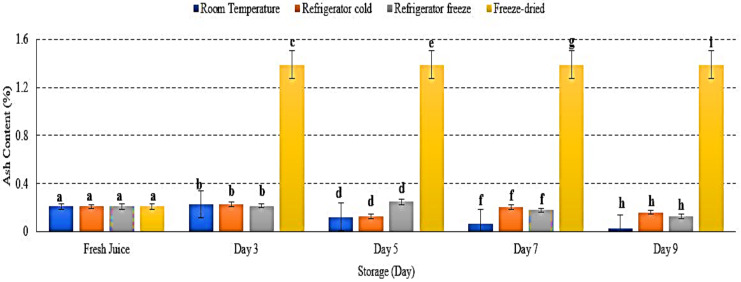
The ash content (%) of watermelon juices during storage at room temperature, refrigerator cold, refrigerator freeze and freeze-dried. Different letters indicate significant differences between samples *p* < 0.01.

The ash content represents the total mineral presented in food [27,28]. There were decreases in ash content in room temperature, refrigerator cold and refrigerator freeze storage, while increased ash content in freeze-dried watermelon juice *p* < 0.05 (Figure 3). The decrease of ash content indicated the susceptibility of ash towards storage condition and its physiological activities, possibly due to the respiration process that decreased the levels of carbohydrate (C), hydrogen (H) and oxygen (O_2_), subsequently decreasing mineral content in samples [29,30]. The study by Tahmasebian et al., 2020 demonstrated the decrease of ash content in probiotic mango juice during storage at 25 °C and further supports the present result [31]. Meanwhile, increased ash content in freeze-dried watermelon juice was due to removal of water during the freeze-drying process, resulting in increased nutrient concentration by fruit’s net weight [32]. Thus, this study demonstrated that storage temperatures and conditions indirectly promote the physiological process and affect ash content in watermelon juice.

### 2.4. Moisture Content (%)

**Figure 4:**
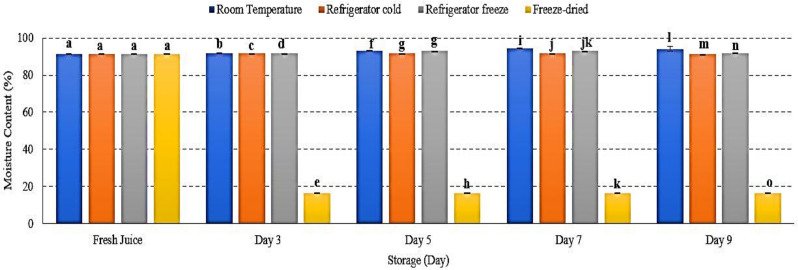
The moisture content of watermelon juice during 9 days of storage. Different letters indicate significant differences between samples *p* < 0.01.

Fresh fruit contains 70–95% of water content [33]. As fruit being stored or processed, moisture content changed and might affected fruit’s quality. The present study demonstrated an increased moisture content (%) of watermelon juice at room temperature and refrigerator freeze, while a decrease in refrigerator cold and freeze-dried storage *p* < 0.01 (Figure 4). The present results indicated that moisture content was influenced by storage temperature and humidity [33,34]. Increased moisture content reflects the permeability of moisture transfer from the surrounding environment into the fruit cells [34,35]. In contrast, a decreased moisture content (%) might be due to decreased surrounding humidity that promotes water loss from the fruit cell as well as the lyophilization process that completely remove the water content during freeze-drying [33,36]. Matured watermelon comprises optimum water content with 93% of fruit net weight [1]. Increased moisture leads to decreased watermelon juice shelf life and develops microbial growth [34]. Hence, this study showed that moisture content in watermelon juice was affected by temperature and humidity of storage conditions that promote the interchange of water content during 9 days of storage.

### 2.5. Total Soluble Solid

**Figure 5:**
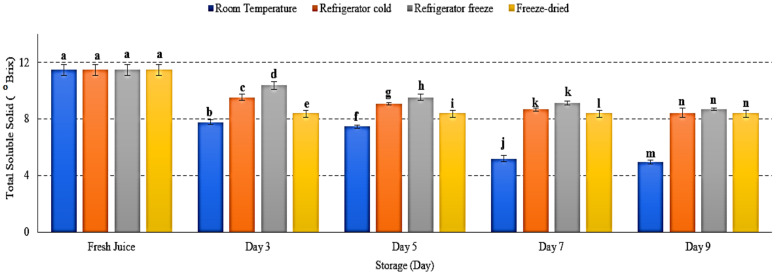
The total soluble solid of watermelon juice during 9 days of storage. Different letters indicate significant differences between samples *p <* 0.05.

Total soluble solid (TSS) was performed to measure the total sugar content presented in fruit juice [37]. There was a decrease of TSS in watermelon juice stored at all storage conditions with *p* < 0.05 (Figure 5). Room temperature displayed the lowest TSS on day 9 of storage. The decrease of soluble solid eventually reduced fruit juice sweetness. The results attributed to enhanced respiration rate occur during storage [38]. Hydrolysis of sucrose and utilization of reducing sugar by microorganism in fruit juice leads to increased production of organic acid, hence reduced TSS and pH [38,39]. Jerry and Bright (2019) further revealed that soluble solids retain longer in low-temperature storage, which supported the present study [40]. Thus, this study showed that temperature may influence fruit sugar content, which will eventually reduce the total soluble solid in juice.

### 2.6. Browning Reaction

**Figure 6:**
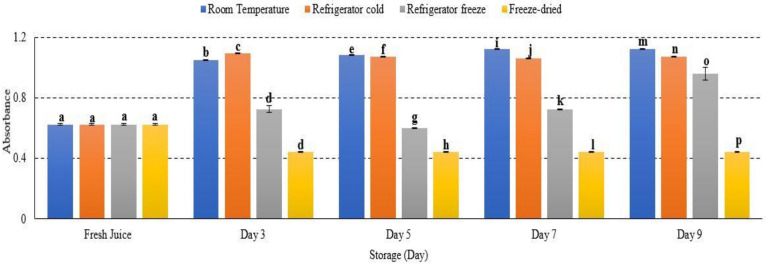
Browning reaction of watermelon juice during storage at different temperature. Different letters indicate significant differences between samples *p* < 0.01.

Most reactions take place during fruit storage, which may affect colour changes such as a browning reaction [41]. There was increased browning reaction in room temperature, refrigerator cold and refrigerator freeze, while decreased browning reaction was observed in freeze-dried watermelon juice with significant differences of *p* < 0.01 (Figure 6). Room temperature displayed the highest browning reaction (Figure 6). An increase in browning reaction of fruit juice during storage is a result of a non-enzymatic process known as the Maillard reaction [25]. During the Maillard reaction, hydroxymethylfurfural (HMF), a product of caramelization (pyrolysis of sugar), forms to cause browning in fruits. The formation of HMF was reported to increase proportionally in increased storage temperature and time [25]. This is in line with a study done by Selli and coworkers (2002), who reported the increase of browning index in the juice of orange wine due to ascorbic acid degradation and increased production of carbonyl compound that eventually induced browning [42]. This outcome was further supported by Donno et al., 2019, in which an increased storage temperature promoted a browning reaction in fruits [43]. In general, a browning reaction often occurred at the end stage of fruit ripening. However, many studies also revealed that the internal browning reaction occurred during exposure towards oxidative stress, temperature and prolonged storage [42,43,44]. Hence, this study demonstrated an increase in HMF concentration in watermelon juice advent to increase temperature storage, resulting in an intense browning reaction. 

### 2.7. Turbidity

**Figure 7:**
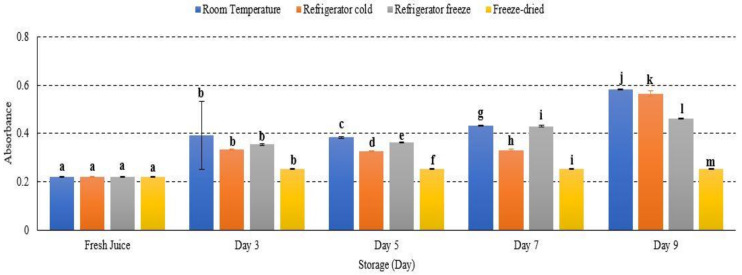
The absorbance turbidity of watermelon juice during storage. Different letters indicate significant differences between samples *p* < 0.05.

Turbidity or cloudiness was used to measure the clarification efficiency of juice sample. This study presented an increased turbidity at room temperature, refrigerator cold, refrigerator freeze and freeze-dried watermelon juice storage with *p* < 0.05 (Figure 7). Room temperature storage contributed the highest turbidity among other storage (Figure 7). The turbidity results are attributed to protein–polyphenol interaction and spoilage from yeast and bacteria, prominently at a higher temperature [45]. Increased temperature storage eventually rises the level of turbidity in fruit juice [46,47]. Moreover, increased standard deviation observed for watermelon juice stored at room temperature (day 3) might be due to instability of haze formation in the juice compound [46]. Thus, this study presented an increased turbidity of watermelon juice advent to increased storage temperature.

### 2.8. Total Phenolic Content (TPC)

**Figure 8:**
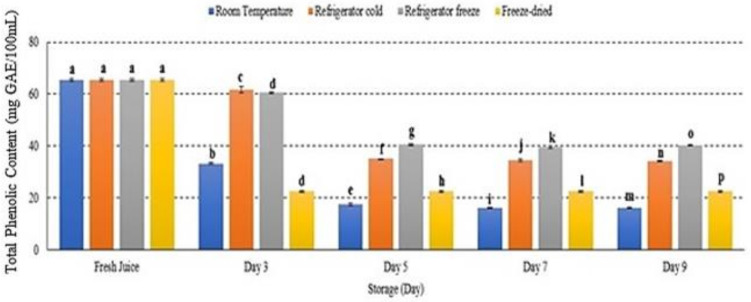
The total phenolic content in watermelon juice. Different letters indicate significant differences between samples *p* < 0.05.

The phenolic compound in watermelon juice decreased at all storage temperatures with significant differences observed *p* < 0.05 (Figure 8). After 9 days of storage, room temperature displayed the highest phenolic degradation among other storage conditions (Figure 8). The present study demonstrated that refrigerator freeze was able to retain phenolic degradation under 9 days of storage period. The study attributed that phenolic degradation was affected by storage temperature, particularly increased storage temperature, and subsequently promoted phenolic degradation [48,49]. Hence, this supported that TPC is temperature-dependent [48]. 

Phenolic compound plays an important role in maintaining human health due to its strong antioxidant potency. The properties have been reported as health-protecting agents that are able to reduce the risk of chronic diseases including cancer and heart disease [50]. However, phenolic compounds are sensitive and could degrade under various environmental conditions such as light, pH, oxygen, temperature and ions [51] due to their unstable structures. Alteration of phenolic structure degrades its antioxidant activities that eventually reduce juice quality and contribute to nutritional loss [51]. Several studies supported the present study outcomes indicating that the degradation of phenolic compounds may be affected by storage temperature [50,52]. For these reasons, storage temperature is an important factor in maintaining phenolic bioactive compounds in watermelon juice. 

### 2.9. Total Flavonoid Content (TFC)

**Figure 9:**
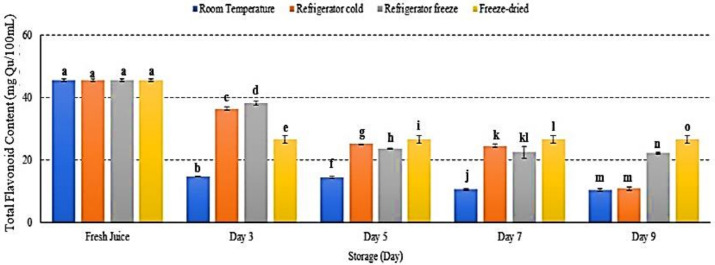
The TFC in watermelon juice during storage. Different letters indicate significant differences between samples *p* < 0.05.

Flavonoids are plant secondary metabolites, which belong to phenolic groups [52]. Interestingly, flavonoids also act as antioxidants in scavenging free radical and reactive oxygen species [53]. The present study showed decreased flavonoid content in watermelon juice stored at all storage temperatures *p* < 0.05 (Figure 9). After 9 days of storage, room temperature and refrigerator cold showed the lowest flavonoid content, followed by refrigerator freeze and freeze-dried storage (Figure 9). Although TFC level in both refrigerator cold and room temperature storages displayed 11 mg/Qu on day 9, the degradation trend of flavonoid compound in refrigerator cold storage was slower compared to room temperature (Figure 9). Phytochemical phenolic and flavonoid compounds are temperature-sensitive and easily deteriorate under various conditions of temperature, light, pH, and oxygen, resulting in loss of its antioxidant and bioavailability activities [54,55]. The results indicated that cold storage temperature retained flavonoid stability [29]. An increased storage temperature was able to promote flavonoid degradation [49] and resulted in alteration of phenylalanine, hence reduced flavonoid content [56]. The present study supported that exposure towards higher temperature altered flavonoid structures, subsequently inducing degradation [29]. Furthermore, the study demonstrated an increased flavonoid in freeze-dried watermelon juice on day 7 and day 9 of storage with decreased phenolic content possibly due to poor selectivity of the Folin Ciocalteu method to quantify total phenolic in the sample extract [57]. Hence, this study presented the loss of TFC in watermelon juice during storage due to alteration of flavonoid structures at storage temperature.

### 2.10. Antioxidant Scavenging Activities Using DPPH

**Figure 10:**
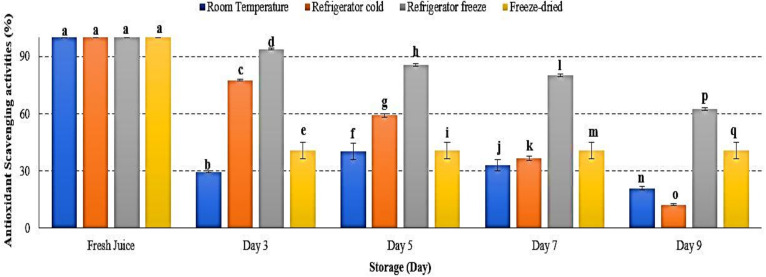
Antioxidant scavenging activities in watermelon juice. Different letters indicate significant differences between samples *p* < 0.05.

Antioxidant is defined as a substrate resulting in delayed, prevented and removed oxidative damage [58]. Antioxidant scavenging activities in watermelon juice were decreased in all storage temperatures, with significant differences observed at *p* < 0.05 (Figure 10). After 9 days of storage, refrigerator cold demonstrated the lowest antioxidant scavenging activities followed by room temperature, freeze-dried and refrigerator cold storage (Figure 10). The present results indicated an increased degradation of antioxidant in watermelon juice during storage. The efficacy of antioxidant activities were too dependent on the storage temperature and time exposure. Antioxidant scavenging activities were reported to be more prominent at a higher storage temperature, indicating that antioxidant scavenging activities decreased at higher storage temperature [59]. Moreover, increased storage temperature promoted the degradation of phenolic and flavonoid compounds, leading to ineffectiveness of its antioxidant scavenge properties [60,61]. The study results revealed that the degradation of antioxidant scavenging activities in watermelon juice during 9 days of storage was affected by storage temperature.

### 2.11. Lycopene and β-Carotene Quantification using HPLC-DAD

**Figure 11:**
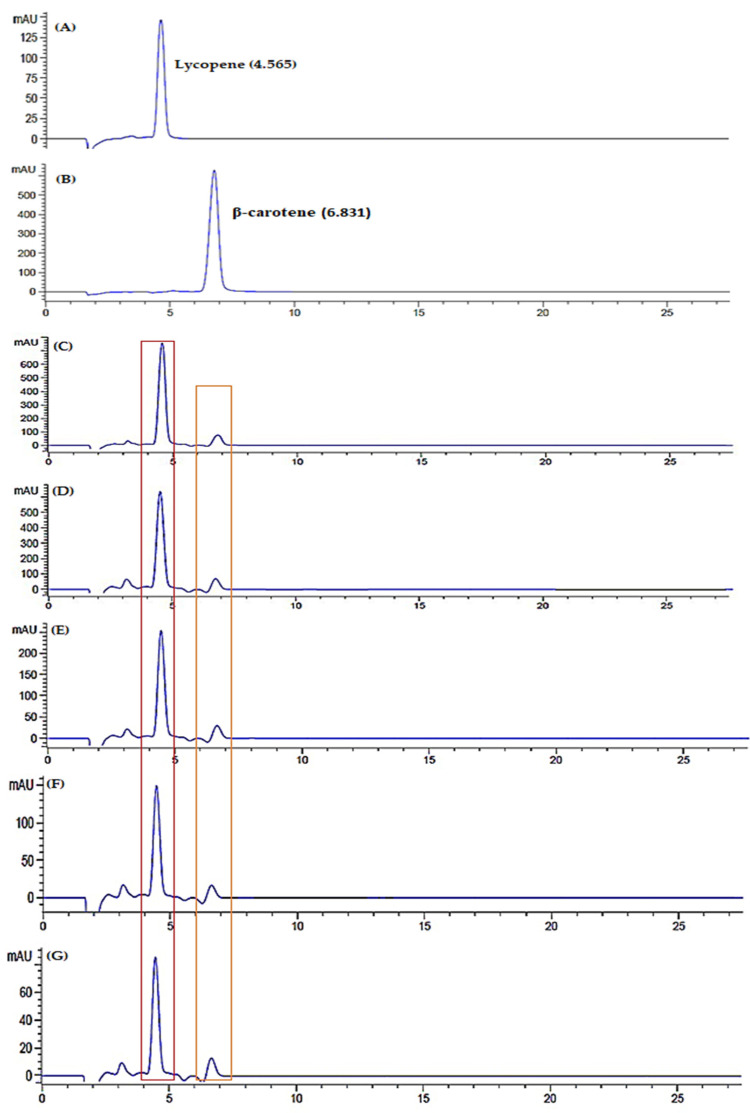

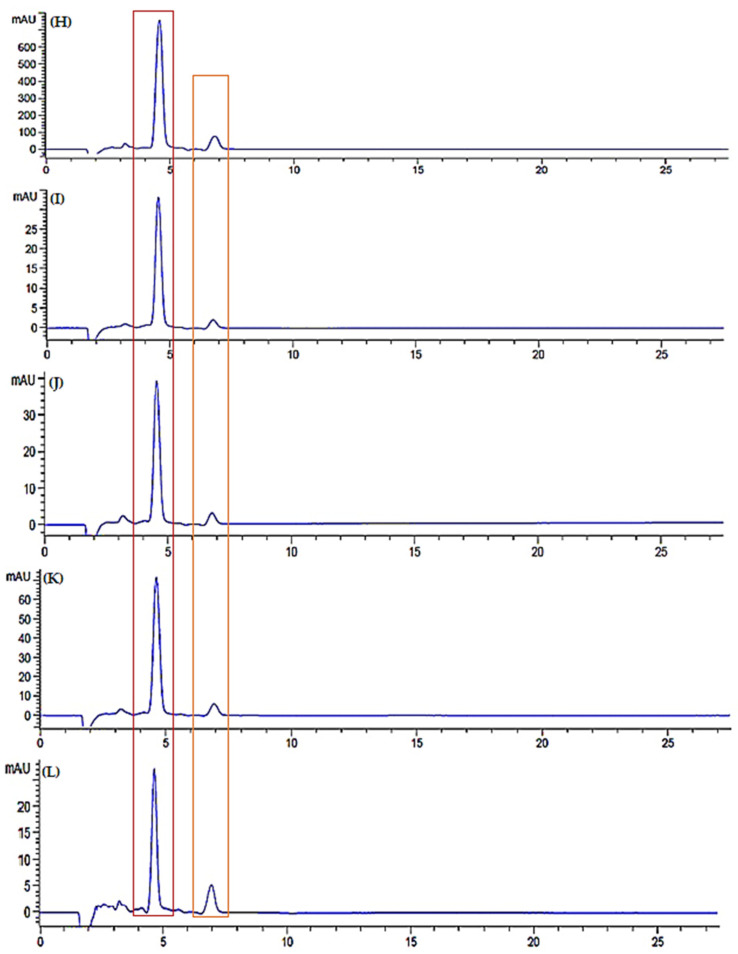

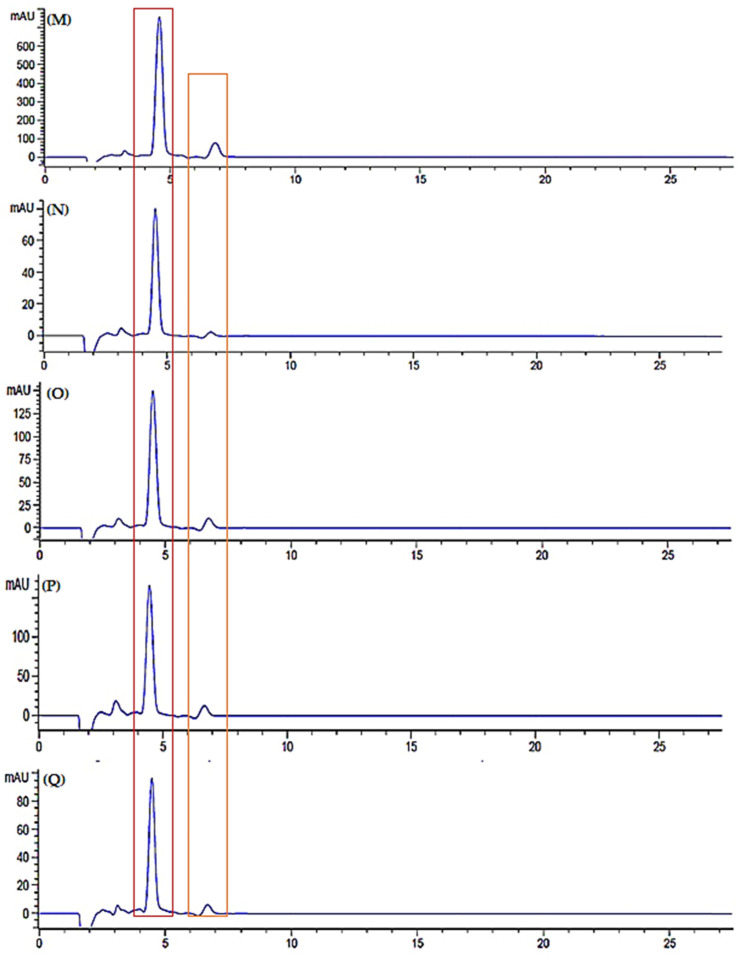

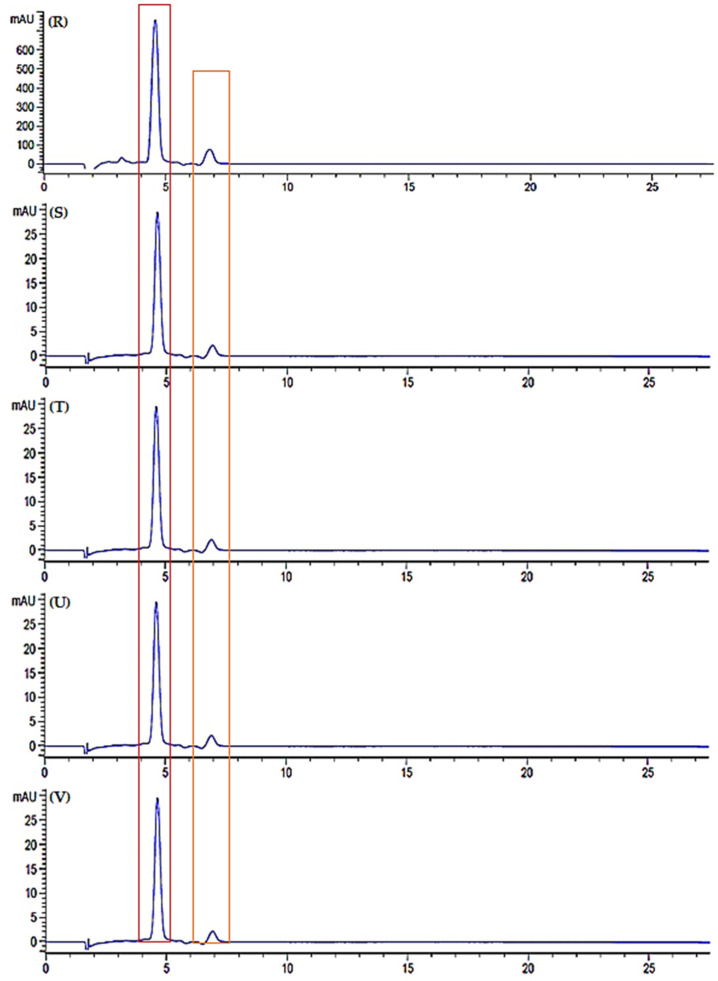
Chromatogram of standard (**A**) lycopene and (**B**) β-carotene, chromatogram of watermelon juice in room temperature storage; fresh juice (**C**), day 3 (**D**), day 5 (**E**), day 7 (**F**) and day 9 (**G**), chromatogram of watermelon juice in refrigerator cold storage; fresh juice (**H**), day 3 (**I**), day 5 (**J**), day 7 (**K**) and day 9 (**L**), chromatogram of watermelon juice in refrigerator freeze storage; fresh juice (**M**), day 3 (**N**), day 5 (**O**), day 7 (**P**) and day 9 (**Q**), chromatogram of freeze-dried watermelon juice; fresh juice (**R**), day 3 (**S**), day 5 (**T**), day 7 (**U**) and day 9 (**V**).

HPLC analysis was performed using C-18 column (150 mm × 4.6 mm), 5 mm paired with diode array detector (DAD) and a flow rate of 1 mL/min of mobile phase acetonitrile:water (95:5). The injection volume of sample used was 20 µL with detection DAD wavelength at 470 nm. Five different concentrations of lycopene and β-carotene standard were injected into HPLC column to plot a calibration curve. From the calibration curve, the regression equation for lycopene and β-carotene were:

y = 1.2029x − 6.5596, R^2^ = 0.9972 (1)

and

y = 5.8788x − 437.57, R^2^ = 0.9955 (2)

respectively. The limit of detection (LOD) and limit of quantification (LOQ) were in the range of 0.30–10.09 µg/mL, with accuracy of 92–103% presented such that the optimized method was precise, sensitive and reliable to simultaneously identify lycopene and β-carotene in watermelon juice using HPLC (Figure 11) [29,62].

**Figure 12:**
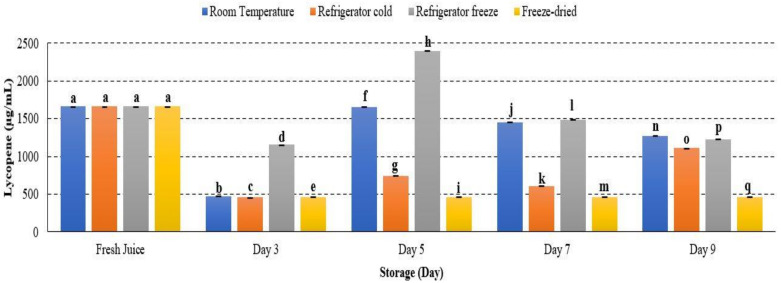
Lycopene quantification in watermelon juice. Different letters indicate significant differences between samples *p* < 0.05.

Lycopene and β-carotene are major carotenoid properties presented in watermelon. Lycopene is responsible for the deep colour pigmentation, while β-carotene lends the orange-yellow watermelon colouration. These carotenoids reflect fruit quality and its antioxidant availability. Lycopene concentration in watermelon juice showed significant differences during storage at room temperature, refrigerator cold, refrigerator freeze and freeze-dried with *p* < 0.05. The concentration of lycopene in watermelon juice decreased on day 3, increased on day 5 and started to decline until day 9 (Figure 12). Patanè et al., 2019 supported the present study, indicating that lycopene stability was affected during the storage period [63]. The initial decrease of lycopene concentration attributed to high susceptibility of active metabolites towards oxidative damage [63]. Moreover, a minor increase of lycopene attributed to the occurrence of remaining lycopene biosynthesis during shelf life, commonly processed at the pink or light-red stage [64]. In addition, lycopene is a deep-red colour pigmentation that is unstable under various environmental conditions. The structure of lycopene that comprises seven double bonds can lead to easily induced degradation. Hence, the decrease of watermelon lycopene occurred until day 9 of storage.

**Figure 13:**
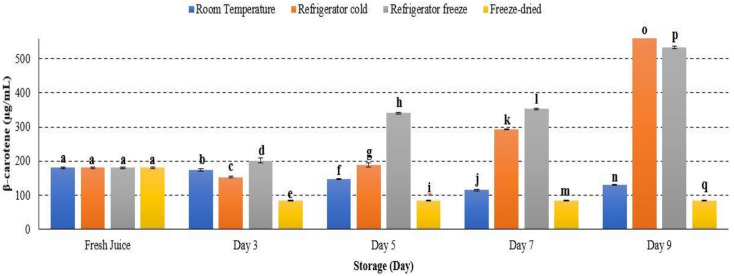
The quantification of β-carotene in watermelon juice. Different letters indicate significant differences between samples *p* < 0.05.

β-carotene concentration increased from day 1 to day 9 of storage at refrigerator cold and refrigerator freeze, while it decreased in room temperature and freeze-dried storage conditions (*p* < 0.05) (Figure 13). The result indicated that β-carotene retained stability in cold storage [23]. The increment of β-carotene concentration during the ripening process can be induced by ethylene. The production of ethylene was reported prominently in cold storage and subsequently supported the present study [64]. Furthermore, β-carotene is thermolabile-, light- and oxygen-sensitive [65], and decreased β-carotene concentration may possibly indicate the maximal degradation of β-carotene occurrence under room temperature storage [66]. A study further described that high storage temperature may inhibit biosynthesis of carotenoids by limiting the production of ethylene and lead to reduced carotenoid precursor [23]. Hence, the present study demonstrated that the ripening enzyme, ethylene, may affect β-carotene concentration in watermelon juice at different temperature storages. 

## 3. Materials and Methods

### 3.1. Chemicals and Reagents 

All solvents were HPLC grade, purchased from Merck (Darmstadt, Germany). HPLC grade of n-hexane, tetrahydrofuran, methanol (MeOH) and acetonitrile (MeCN) were purchased from Merck Millipore (Darmstadt, Germany). Analytical grade powder of sucrose (≥99.5%), gallic acid, quercetin, ascorbic acid, DPPH, lycopene (≥98%) and β-carotene (≥95%) were purchased from Sigma-Aldrich (St. Louis, MO, USA). 

### 3.2. Watermelon Collection

Round-to-oval-shaped watermelons (*Citrullus lanatus)* ranging from 2.2 kg to 3.2 kg, harvested in 2021, were obtained from a local farm located in Subang, Selangor, Malaysia. The sampling of watermelon fruits was carried out in the morning. The fruits were selected based on similarity of size, maturity and colour. Damaged and unshaped watermelon fruits were removed from the sampling. Watermelon samples were sent to Forest Research Institute Malaysia (FRIM) for species identification with reference no: FRIM700-1/1/1Kit.3(87). 

### 3.3. Preparation of Watermelon Juice

Watermelons were washed and wiped with tissue papers. Then, they were peeled, weighed, and cut before being homogenized into juice using a juice mixer (Panasonic Classic Series Juice Extractor PJ-67, Panasonic Corporation, Osaka, Japan). The juice was made into aliquot, filled into a 50 mL falcon tube bottle, and wrapped with aluminium foil to reduce light exposure during storage. Approximately 400 mL (8 falcon tubes) of watermelon juice were separated and stored at room temperature (25 °C), refrigerator cold (4 °C), refrigerator freeze (−8 °C) and freeze at −80 °C, which later underwent a freeze-dried process in a freeze-dryer at 0.340 mBar at a temperature of −47 °C for three days (LabQuip freeze-dryer, Selangor, Malaysia). All watermelon juice at different storage conditions were analyzed on day 1, 3, 5, 7, and 9.

### 3.4. Weight Loss

For determining weight loss, watermelon fruit samples stored at different storage temperatures were calculated for their primary and secondary weight differences [10]. The weight loss of watermelon was expressed as a percentage weight loss, as follows:

(3) $$\mathrm{Weight}\;\mathrm{loss}\;\left(\%\right)=\frac{\mathrm{Primary}\;\mathrm{weight}-\mathrm{secondary}\;\mathrm{weight}}{\mathrm{Primary}\;\mathrm{weight}}\times 100$$

### 3.5. pH

The pH of watermelon juice was determined by using pH meter (Knick pH-Meter 765 Calimatic, Berlin, Germany). Watermelon juice with a volume of 20 mL was placed in a beaker and the pH of each juice sample was recorded in triplicate, as referred to Sabeetha et al., 2017 [7].

### 3.6. Ash Content (%)

The ash content (%) of watermelon juice was performed, as referred to Okokon and Okokon (2019) [67]. An empty crucible was weight (W1) using electronic analytical balance. Then, 10 mg of watermelon juice sample were weighed in the empty crucible (W3). The watermelon juice sample in the crucible was then placed in a muffle furnace (Type 30400, Barnstead Thermolyne Corporation, Dubuque, IA, USA) at 550 °C for 6 h or until whiteish grey ash formed. The crucible was then placed in a desiccator to let it cool, then weighed (W2). The ash content in watermelon juice was then calculated as follows:

(4) $$\mathrm{Ash}\;\mathrm{content}\;\left(\%\right)=\frac{W2-W1}{W3}\;\times \;100$$

where, W1 = weight of empty crucible; W2 = weight of crucible with ash; W3 = weight of watermelon juice sample.

### 3.7. Moisture Content (%)

The moisture content (%) of watermelon juice was determined according to the methods performed by Sabeetha et al., 2017 and Okokon and Okokon (2019) [7,67]. An empty crucible was weight (W1) using electronic analytical weighting balance. Then, approximately 10 mg of watermelon juice sample were weighed in the empty crucible (W3). The watermelon juice sample in the crucible was then placed in a drying oven at a temperature of 105 °C for 6 h or until the watermelon juice sample completely dried. The crucible was later being placed in a desiccator to let it cool, then weight (W2). The percentage of moisture content was then calculated using the following equation:

(5) $$\mathrm{Moisture}\;\mathrm{content}\;\left(\%\right)=\frac{W2-W1}{W3}\;\times \;100$$

where W1 = weight of empty crucible; W2 = weight of crucible with dried sample; W3 = weight of watermelon juice sample.

### 3.8. Total Soluble Solid 

Total soluble solid (TSS) of watermelon juices were determined using a hand-held refractometer as performed by Sabeetha et al., 2017 [7]. The TTS in watermelon juice was expressed as °Brix. Approximately 2 drops of juice samples were placed on the clear prism of refractometer. The °Brix value of each watermelon juice was recorded in triplicate. Refractometer was calibrated using sucrose solution 10% for each usage.

### 3.9. Browning Reaction 

Browning of watermelon juice was evaluated by the measurement of absorbance at 420 nm as performed by Paravisini and Peterson (2018) [68]. Approximately 1 mL of watermelon juice was centrifuged at 3000 RPM for 10 min. The juice supernatant (1000 µL) was then transferred into a cuvette and directly assessed on its browning reaction using UV-Visible spectrophotometer (UV-1800, Shimatzu Corporation, Kyoto, Japan) at 420 nm against water. The absorbance measurement of each watermelon juice was performed in triplicate.

### 3.10. Turbidity

Turbidity or cloudiness of watermelon juice was evaluated as referred to Queirós et al., 2014 [15]. Watermelon juice was centrifuged at 3000 RPM for 10 min and inserted in a cuvette. The absorbance measurement was measured using UV-Visible spectrophotometer (UV-1800, Shimatzu Corporation, Kyoto, Japan) at wavelength 700 nm against water. The absorbance measurement was performed triplicate.

### 3.11. Total Phenolic Content (TPC)

TPC was determined using the Folin–Ciocalteu method as referred to Amini et al., 2017 [69]. Approximately 1 mL of juice sample was added into the labelling test tube containing 1 mL of aqueous 10% Folin–Ciocalteu’s reagent. The mixtures were shaken well and incubated in room temperature for 6 min. Later, 3 mL 7.5% sodium carbonate solution (Na_2_CO_3_) in water were added and incubated at room temperature for 60 min. The absorbance of juice mixture was measured using UV-Visible spectrophotometer (UV-1800, Shimatzu Corporation, Kyoto, Japan) at 765 nm against blank. Blank sample was prepared by mixing 1 mL of distilled water, 1 mL of Folin–Ciocalteu reagent and 3 mL of 7.5% Na_2_CO_3_ aqueous solution. Gallic acid was used as a standard. The total phenolic content in watermelon juice was expressed as mg of gallic acid equivalent per volume of sample (mg GAE/100 mL).

### 3.12. Total Flavonoid Content (TFC)

TFC was measured by adding 2 mL of 2% aluminium trichloride (AlCl_3_) in methanol, into a labelling test tube containing 2 mL of watermelon juice. The mixtures were shaken well and incubated in a dark room temperature for 30 min. After the incubation period, absorbance was measured using UV-Visible spectrophotometer (UV-1800, Shimatzu Corporation, Kyoto, Japan) at 415 nm against blank. Blank sample was prepared by mixing 2 mL of juice sample with 2 mL of methanol without the addition of aluminium trichloride. The quantification of total flavonoid content in the watermelon juice was referred on quercetin standard curve and the results obtained were expressed as milligrams of quercetin equivalent per volume of sample (mg Qu/100 mL) [69].

### 3.13. 2,2-1-Diphenyl-1-picrylhydrazyl (DPPH)

DPPH was used to measure the antioxidant scavenging activities in watermelon juice during storage as referred to Amini et al., 2017 [69]. Watermelon juice with volume of 4 mL was added into a clean test tube containing 0.5 mL of DPPH (0.5 mM) in methanol. The mixture was mixed and incubated in dark room temperature for 30 min. After the incubation period, the absorbance of antioxidant scavenging activities was measured using spectrophotometer (UV-1800, Shimatzu Corporation, Kyoto, Japan) at 517 nm against blank. In this assay, methanol was used as a blank. For positive control preparation, 4 mL of methanol were mixed with 0.5 mL of 0.5 mM DPPH solution and later measured under 517 nm wavelength. Ascorbic acid solution was used to obtain a standard calibration curve. The radical scavenging activities of ascorbic acid standard and watermelon juice were calculated using the equation below and were expressed as percentage antioxidant scavenging activities.

(6) $$\mathrm{Antioxidant}\;\mathrm{scavenging}\;\mathrm{activities}\;\left(\%\right)=\frac{\mathrm{Ac}-\mathrm{As}}{\mathrm{Ac}}\times 100$$

where Ac is the absorbance of control and As is the absorbance of standard or sample.

### 3.14. Extraction Procedure

The extraction procedure was performed to extract lycopene and β-carotene from watermelon juice prior to high-performance liquid chromatography (HPLC) analysis. Approximately 100 mL of watermelon juice were extract with 100 mL of n-hexane for 24 h on a reciprocating shaker (180 rpm) (IKA KS 130 basic, Staufen, Germany). The extraction juice was later transferred into a centrifuge tube and centrifuged at 3000 rpm for 10 min. The centrifugation step resulted in orange-yellowish n-hexane supernatant consisting of interest lycopene and β-carotene (upper layer). The upper-layer supernatant was separated and evaporated using rotary evaporator (Buchi R 210, Flawil, Switzerland) in order to obtain crude sample and remove n-hexane. The residue of watermelon juice (bottom layer) later was repeated twice for the extraction with n-hexane to completely extract lycopene and β-carotene. Crude watermelon juice obtained was kept in −80 °C prior to analysis.

### 3.15. Sample and Standard Preparation for HPLC

Lycopene and β-carotene standards (1 mg) were dissolved in tetrahydrofuran (0.5 mL) and methanol (0.5 mL) to obtain stock solution of 1000 µg/mL and later filtered through a 0.45-µm polyvinylidene fluoride (PVDF) syringe filter into 2 mL of amber HPLC vial. Approximately 500 µg/mL of extract sample were prepared by dissolving 0.5 mg of watermelon juice extract in 1 mL of tetrahydrofuran and methanol (1:1). The extract solution was then filtered through a 0.45-µm polyvinylidene fluoride (PVDF) syringe filter into 2 mL of amber HPLC vial to reduce light exposure.

### 3.16. HPLC-DAD Analysis of Lycopene and β-Carotene

The identification and quantification of lycopene and β-carotene in watermelon juice were analyzed by using high-performance liquid chromatography-diode array detector (HPLC-DAD) as referred to Noh et al., 2020 with slight modifications [6]. Agilent HPLC (1200 Agilent, Santa Clara, CA, USA) equipped with auto-sampler injector (G1328B), column oven, quat pump (G1311A), degasser (G1311A) and diode array detector (DAD) were used for the analysis. Twenty microliters of each standard and sample were injected into ZORBAX SB-C18 column, 5µm (4.6 × 150 mm) (Agilent, Santa Clara, CA, USA) at maintained temperature of 45 °C using isocratic mode of HPLC mobile phase acetonitrile and water (95:5, *v/v*) with flow rate of 1 mL/min. The separation and detection of lycopene and β-carotene in standard and sample were carried out in triplicate into C-18 column coupled with diode array detector at wavelength 470 nm. 

Quantitative analysis was carried out using a calibration curve of standard and the result was expressed in microgram per milliliter of juice (µg/mL). The validation of HPLC method was referred to the International Conference of Harmonisation (ICH) using linearity, limit of detection (LOD), limit of quantification (LOQ) and accuracy. The linearity of HPLC method was constructed using the concentration range of 31.25–1000 µg/mL for lycopene and β-carotene. The regression equation was calculated in the form of

y = ax + b (7)

where x is the concentration of standard and y is the peak area of the interest compound. Linearity was established by determination of regression coefficient (R^2^) value. LOD is the lowest concentration of analyte that can be detected while LOQ is the concentration of analyte that can be measured quantitatively. LOD was detected using a single-to-noise ratio at 3:1 while LOQ was calculated at 10:1. Precision of the method was performed by determining the relative standard deviation (RSD) for intraday (three times analysis in one day) and interday (three days analysis). Accuracy of the method was performed using recovery study by injecting a known amount of standard solution (three concentrations) into the test samples.

### 3.17. Statistical Analysis 

All analyses were performed in triplicate and the results were expressed as mean values ± standard deviation. One-way ANOVA was used for analyzing the data with 95% confident interval (*p* < 0.05) indicated significant changes. Duncan’s Multiple Range Test was used to analyze the changes of watermelon juice at different storage temperature (SPSS Inc., Chicago, IL, USA).

## 4. Conclusions

This study indicated that watermelon juice undergoes degradation of physical and chemical properties advent to storage temperature which later reduced juice quality and nutritional values. There were decreased quality of watermelon juice in all storage temperatures. However, the degradation trend differed according to storage temperatures and conditions. The results showed that the best storage of watermelon juice is in sequence of freeze-dried > refrigerator freeze > refrigerator cold > room temperature. The consumption of fresh watermelon juice was notable beneficial to health. However, freeze-dried might be used for watermelon juice storage as it is able to retain nutritional value of phytochemicals; phenolics, flavonoids, carotenoids and antioxidant as well as delayed juice degradation. The outcomes of the present works provide an effective way for watermelon juice storage, but further research required to obtain the kinetic shelf-life of watermelon juice under respective conditions.

## Data Availability

The data presented in this study are available on request from corresponding author. The data are not publicly available due to being part of a research project.
